# Experimental Study on Performance Optimization of Grouting Backfill Material Based on Mechanically Ground Coal Gangue Utilizing Urea and Quicklime

**DOI:** 10.3390/ma16031097

**Published:** 2023-01-27

**Authors:** Xiao Wang, Jixiong Zhang, Meng Li, Binbin Huo, Ling Jin

**Affiliations:** 1 School of Mines, China University of Mining and Technology, Xuzhou 221116, China; 2 State Key Laboratory of Coal Resources and Safe Mining, China University of Mining and Technology, Xuzhou 221116, China

**Keywords:** grouting backfill, coal gangue, ball grinding, transportability, compressive strength

## Abstract

Previously conducted studies have established that grouting backfill in mining-induced overburden bed separation and mined-out areas with broken rocks provides an efficient strategy to control strata movement and surface subsidence caused by underground mining. Grouting backfill materials (GBMs) based on coal gangue (CG) are highly desirable in coal mining for accessibility to abundant CG and urgent demand for CG disposal. However, CG is generally employed as coarse aggregate due to rather rigid and inert properties, limiting its application in GBMs. Herein, to reduce reliance on fine aggregates, such as fly ash and clay, cemented GBM formulations using ground CG powder as a dominant component were proposed. Urea and quicklime were utilized as additives to optimize slurry transportability and compressive strength. Besides typical grinding without additives, CG powder was also prepared via grinding with urea, intending to enhance the hydrogen bonding (HB) interaction between urea and minerals contained in CG. The effect of grinding time and urea on CG particle size and phase composition was investigated. Then, the dependence of slurry transportability and compressive strength on grinding time, solid concentration, urea, and quicklime dosage were revealed. It has been experimentally proved that grinding for 30~90 min significantly decreased CG particle size and even induced crystal deformation of dolomite and kaolinite. For GBMs, urea improved slurry flowability, possibly caused by decreased water absorption on the CG surface and the release of water encapsulated in hydrated cement particles. Moreover, quicklime strengthened GBM bodies, which could be explained by an accelerated pozzolanic reaction between CG powder and additional CH supplied by quicklime hydration. G60U3-based GBM-B2 with 5% quicklime provided a stable and smooth slurry with a bleeding rate of 1.25%, a slump flow of 205 mm, and a hardened body with a seven-day UCS of 1.51 MPa.

## 1. Introduction

Strata movement induced by underground mining leads to serious issues, such as surface subsidence [1], underground roof fall [2], and water loss [3]. As a green mining technique, grouting backfill is developed to fill the mining-induced overburden bed separation and mined-out area with caving rocks to control the strata movement [4,5,6]. In engineering applications, the raw materials are mixed with water and conveniently transported to the filling site as slurries via a pipe equipped with a pump [4]. The compacted body is finally formed via bleeding water and consolidation of grouting slurry to offer support in the filling site. Typically, powder materials or fine aggregate, such as fly ash (FA) [7,8], clay [9], and sand [10], are utilized with cement to produce stable and uniform GBM slurry, which is benefited from their low cost and superior performance of fluidity.

Coal gangue (CG) is a solid waste generated during coal mining and washing, which accounts for approximately 15% of coal production [11]. Currently, the accumulated gangue exceeds six billion tons in China, and the annual increase is about 500~800 million tons [12]. The incorporation of solid waste into underground backfill provides an efficient strategy for the waste utilization and disposal [12,13,14], which minimizes problems caused by its exposure to the environment [11,15]. Grouting backfill materials (GBMs) based on coal gangue (CG) are highly desirable in coal mining in terms of their accessibility to abundant CG and the urgent demand for utilization and disposal of existing and continuously generated CG [11]. However, CG is generally employed as coarse aggregate in backfilling applications due to its rather rigid and inert properties [16,17,18], limiting its application in GBMs.

The potential of replacing powder materials, such as fly ash, with activated CG obtained by calcination and mechanical grinding to prepare geopolymer and supplementary cementitious materials (SCMs) has been demonstrated [19,20,21]. Compared to calcination at 500~1100 ℃ [11], mechanical grinding in dry or wet conditions under ambient temperature is more applicable to backfill production in coal mines due to lower energy consumption, less release of polluting gas, well-established ball mill equipment, and technology [19,22,23]. The CG particles are crushed into microparticles by a collision between CG and grinding balls, leading to size reduction and an increase in specific surface area (SSA). Moreover, incorporating an alkali activator further facilitates their dissolution and pozzolanic reaction [24,25,26].

For grouting materials, the slurry is supposed to exhibit good workability for convenient transport to target the filling position [27]. Excess water is usually needed to improve transportability but resulting in drainage increase and concretion rate decrease [28,29]. Alternatively, the addition of a chemical superplasticizer (SP) helps to reduce water demand for a better workability [28,30]. Numerous efforts have been made to reveal the plasticizing mechanism, enrich the structural design, and investigate their effects on the hydration and strength [31,32,33]. A reference [34] found that the polyacrylamide SP significantly influenced the workability of cemented paste backfill slurry but retarded the hydration reaction. A reference [22] reported that the CG treated via wet-grind with a polycarboxylate-based SP exhibited obvious improvement in pozzolanic reactivity. However, some particular SPs are not readily available nearby the coal mines.

Recently, the urea, potentially obtained from human urine, was reported to act as an effective SP for 3D printing of lunar geopolymer mixtures to attain the required workability of extrusion and shape retention [35]. Besides, urea was also used to improve the rheological behavior of the kaolinite [36], which is a clay mineral commonly contained in CG [25]. Resembling the amide structure of polyacrylamide SP [34], the urea molecule has a simple structure with one carbonyl group (-CO-) and two amino (-NH_2_) groups, providing both hydrogen bonding (HB) acceptor and donor [37]. Therefore, urea is capable of mediating the HB interaction among water, cement, and minerals. Commercial urea has been widely used as an agricultural fertilizer, industrial chemical, and additive in the daily commodities [38,39,40]. Given the strong mediating effect on HB interaction and widespread accessibility of urea, we envision that incorporating the urea during the mechanical grinding of CG would provide an alternative approach to modify the properties of CG-based grouting materials.

Analyzing the above, it can be noted that performance optimization of backfill materials based on mining waste and additives is a very topical issue. Therefore, the purpose of this study is to provide an optimal GBM formulation based on CG with common additives. It is expected to reduce the reliance of GBM on fine aggregates, such as fly ash and clay, which are possibly not readily available near coal mines. Herein, the cemented GBMs utilizing mechanically ground CG as a principal content are studied. Urea and quicklime were utilized as additives to optimize slurry transportability and compressive strength. In comparison with typical grinding without additives, CG powder was also prepared via grinding with urea, intending to enhance the hydrogen bonding (HB) interaction between urea and minerals contained in CG. Meanwhile, quicklime (CaO) was employed to offer additional calcium hydroxide (CH) for the pozzolanic reaction of ground CG. Hence, this study enriches the GBM formulation based on mechanically ground CG and is expected to provide an alternative to those relying heavily on fine aggregates, such as FA, clay, or cement.

## 2. Materials and Methods

### 2.1. Materials

The coal gangue (CG) from Xinjulong Coal Mine in Shandong Province was utilized as aggregate for grouting backfill materials. Composite Portland cement (PC 42.5) purchased from China United Cement (CUCC) was used as the binder. In backfilling applications, due to the limitation of the filling cost [41], the cement dosage will not be adjusted in a broad range under the premise of ensuring the required strength. Hence, the mass ratio of CG to cement was fixed at 85:15 in this study. Urea (≥99.0%) and quicklime (CaO, ≥98.0%), commercially obtained from Shanghai Titan Technology Co. Ltd., were employed as additives. Tap water with a pH of 7.3 from Xuzhou in Jiangsu was used to mix the solid materials.

### 2.2. Research Protocol of CG-Based Grouting Backfill Materials (GBMs)

**Figure 1:**
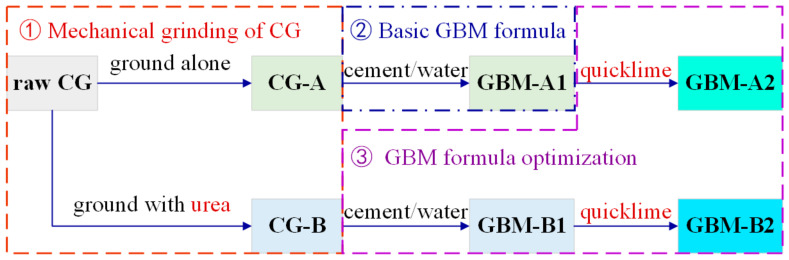
Research process of CG-based grouting backfill materials.

As depicted in Figure 1, the development process of CG-based GBMs underwent three stages. First, mechanical grinding of CG was conducted to change its size and structural characteristics. The effects of milling time and urea dosage on the size distribution and chemical composition were investigated. Then, the CG powder obtained by mechanical grinding without any additives was mixed with cement and water to provide a basic GBM formula. The transportability and strength were studied to evaluate their performance. The mix ratio of raw materials is listed in Table 1. In total, 27 ratios were set, so 81 (27 × 3) samples were prepared separately for bleeding, slump, and UCS tests. Based on the initial GBM formula, the quicklime and CG powder prepared by grinding with urea were utilized to further optimize the slurry transportability and compressive strength of hardened GBMs. The optimal ratio and regulating rules of CBM workability and strength were finally afforded.

### 2.3. Materials Preparation

#### 2.3.1. Grinding Procedure of CG

The raw CG was crushed and sieved through a #80 mesh, providing CG powder for the further grinding process. Grinding of CG was conducted in a BM6Pro planetary ball mill. Zirconia balls of different diameters were employed to provide a well-graded grinding medium, with a weight ratio of 7 mm:5 mm:3 mm = 3:3:4. The zirconia balls (300 g) and CG (100 g) were mixed in the pot and then ground for 30, 60, and 90 min with a speed of 400 r/min. The CG samples milled for 0, 30, 60, and 90 min were labeled as G0, G30, G60, and G90, respectively.

Moreover, to enhance the HB interaction between urea and CG minerals, CG grinding in the presence of urea was also conducted. Laboratory trials of workability and buildability in a reference [35] showed that the urea dosage corresponding to 3% of the regolith mass was optimal. Therefore, in this work, the urea was added with a mass ratio of 1%, 3%, and 5% to CG. The CG samples milled with urea for 30, 60, and 90 min were labeled as GxUy, while x and y correspondingly represented the grinding time (min) and urea dosage (%). For example, G30U1 refers to the CG sample obtained after milling for 30 min with 1% urea.

#### 2.3.2. Preparation of GBM Specimens

According to the predetermined ratio, the CG powder, cement, and additives were mixed with water in a plastic beaker. Then the mixture was stirred at a speed of 200 rpm with a hand-held beater for 5 min, providing GBM slurry series. Urea and quicklime were used as additives are components with minimal amounts. Urea is a water-soluble chemical and grounds extensively with CG, so good homogeneity of urea could be achieved in the preparation of GBMs. Given the hydration of quicklime and low solubility of generated CH, the quicklime was first mixed with water to attain good homogeneity of generated CH in the GBM slurry. For the transportability study, the fresh slurry was used directly. While for the UCS test, the slurry was subsequently poured into a cylindrical mould (with a diameter of 50 mm and a height of 100 mm). After curing in a standard curing chamber at 20 ℃ and 95% RH for seven days, the hardened specimens were finally obtained.

### 2.4. Characterization Methods

#### 2.4.1. Size Distribution and Chemical Composition of CG

The size distributions of CG powder before and after mechanical grinding were tested on a Jinan Winner 3009B laser particle sizer in a range from 0.1 μm to 1200 μm. A bundled Winner software was used to control the test process and provide size reports.

The phase compositions of CG powder before and after mechanical grinding were analyzed by X-ray diffraction (XRD) spectra. A Bruker D8 Advance was used to conduct the XRD tests from 4 to 70°. MDI Jade 6 software was used to interpret the XRD patterns.

The molecular bonds of CG powder before and after mechanical grinding were detected by Fourier transform infrared (FTIR) spectra. A Bruker Vertex 80V spectrometer with a spectral range of 4000~350 cm^−1^ and a resolution of 0.06 cm^−1^ was used to perform the FTIR tests.

#### 2.4.2. Bleeding Rate and Slump Flow of GBM Slurry

The bleeding rate and slump flow were tested to evaluate the transportability of the GBM slurry. The Chinese standard GB/T 50080-2016 was adopted to conduct the bleeding test [28]. The fresh slurry was transferred into a graduated cylinder and placed in a still condition. The bleeding water was steadily sucked out from the cylinder and weighed in 2 h. Finally, the bleeding rate was calculated by the mass ratio of bleeding water to added water in each slurry sample.

The Chinese standard GB/T 8077-2012 was adopted to conduct the slump test [9,42]. The fresh slurry was poured into a circular truncated cone with an upper diameter of 36 mm, a bottom diameter of 60 mm, and a height of 60 mm, which was placed on a smooth plastic plate. The truncated cone was lifted slowly to allow the slurry to flow freely. Typically, the GBM slurry demands for excellent flowability, while the slump is rather large and cannot accurately reflect the flow state. Therefore, the slump flow determined by the average diameter of the slump collapse was used to characterize its fluidity.

#### 2.4.3. Surface Tension of Aqueous Solution

The surface tension of deionized water and urea solution was tested using the ring tear-off method. A Force Tensiometer (K100, KRÜSS GmbH, Hamburg, Germany) was employed to perform the surface tension tests.

#### 2.4.4. UCS of Hardened GBM Specimen

The Chinese standard GB/T 50081-2019 was adopted to conduct the UCS test. The uniaxial compressive strength (UCS) tests were conducted on an MTS WAW-1000D electro-hydraulic servo-controlled test machine. The loading rate was set as 0.5 mm/min. The UCS values were calculated by the ratio of peak stress to the cross-sectional area of tested specimens.

## 3. Results and Discussion

### 3.1. CG Structural Transitions during Mechanical Grinding

#### 3.1.1. Size Distribution

**Figure 2:**
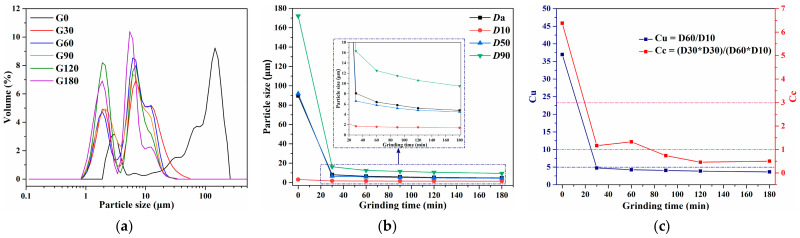
Particle size distribution (**a**), characteristic particle parameters (**b**), Cu and Cc (**c**) of G0 and Gx. D10, D30, D50, D60, and D90 represent the diameter when the cumulative distribution reaches 10, 30, 50, 60, and 90%, respectively.

The particle size characteristics of CG powder ground for 0, 30, 60, 90, 120, and 180 min were first investigated. The size evolutions with grinding time are depicted in Figure 2, which indicates that mechanical grinding significantly decreases particle size. As shown in Figure 2a, a typical bimodal distribution with main ranges of 0.8–2 and 4–20 μm gradually appeared. From Figure 2b, only after grinding for 30 min, the diameter when cumulative distribution reached 90% (D90) declined from 172.2 to 16.3 μm, while the average diameter (Da) declined from 89.6 to 8.1 μm, approximately by 91%. Compared to CG ground for 30 min, the Da reduction of CG ground for 60 min decreased to 21%. With the further increase of grinding duration, the Da continued to decrease slowly by 9%, 10%, and 8% after grinding for 90, 120, and 180 min. Therefore, the refining effect on CG particle size gradually weakened with the grinding duration, especially longer than 90 min. Given the demand for reducing energy consumption, CG powder grounding for no longer than 90 min was mainly studied in a subsequent study.

The nonuniformity coefficient (Cu) and the curvature coefficient (Cc) were reported to evaluate the size uniformity and gradation. Hence, Cu and Cc evolution are further studied and displayed in Figure 2c. The continuous decrease of Cu (blue curve) from 37 to lower than 5 proved that the size distribution of CG particles obviously narrowed with grinding. For CG directly obtained via sieving without further grinding, the Cc of 6.4 indicated that it lacked particles between D10 and D30. After grinding for 30 and 60 min, CG with the Cc between 1 and 3 resulted in rather well-graded distributions. After grinding for 120, 150, and 180 min, the Cc lower than 1 implied that the CG lacked particles between D30 and D60.

**Figure 3:**
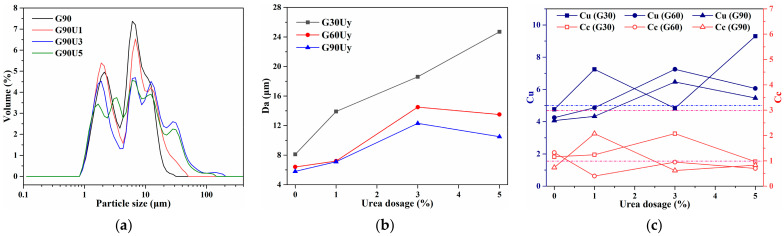
Particle size distribution (**a**), average diameter Da (**b**), Cu and Cc (**c**) of Gx and GxUy.

Given the mediating effect of urea on HB interaction, CG grinding in the presence of urea was also conducted to enhance the HB interaction between urea and CG minerals. Urea was added prior to grinding with 1%, 3%, and 5%, determined by the mass ratio of urea to CG. The effect of urea dosage on the size distribution of CG was investigated. The size characteristics with different grinding times and urea dosages are displayed in Figure 3. The size distribution in Figure 3a shows that, with the urea dosage increasing from 0~1% to 3~5%, multiple distributions with a main range of 0.8–100 μm instead of the typical bimodal distribution appeared. Da trends in Figure 3b proved that Da of CG increased from 5.8~8.1 μm to 10.5~24.7 μm with the urea dosage varying from 0 to 1~5%. Therefore, the results indicate that, compared with CG obtained by grinding without any additives, the presence of urea tends to weaken the refining effect of mechanical grinding on CG particle size. Moreover, in accordance with the multiple distributions in Figure 3a, Cu evolution in Figure 3c also suggested the increase in the nonuniformity of CG particles with urea addition. For CG powder obtained by grinding for 30 min with 1% urea, the Cu higher than 5, as well as Cc between 1 and 3, indicated a typical well-graded distribution.

#### 3.1.2. Phase Component

**Figure 4:**
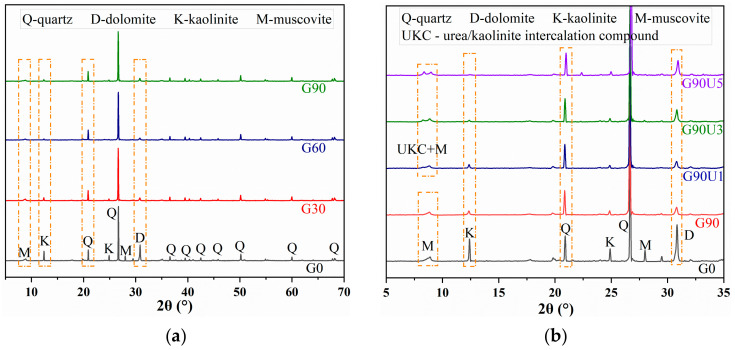
XRD spectra of Gx (**a**) and GxUy (**b**).

XRD spectra were utilized to characterize the mineralogical phase composition of CG. Figure 4 shows the transformation in the phase component of CG caused by mechanical grinding. For raw CG (black line) in Figure 4a, quartz, dolomite, kaolinite, and muscovite were identified as primary mineralogical components. After grinding for 30, 60, and 90 min, a vast amount of quartz still remains, mainly due to its rather stable silicon–oxygen tetrahedron structure [25]. Meanwhile, the intensity decreased in the feature diffraction peak of dolomite, kaolinite, and muscovite, providing evidence for the obvious destruction of their crystal structures. Dolomite [CaMg(CO_3_)_2_] is a carbonate mineral typically used as a calcium and magnesium supplement [43]. It was reported that the unit cell of dolomite remained intact even after grinding for 24 h. However, an irreversible strain was introduced into the crystal structure [44]. Moreover, studies on wet-milling of dolomite proved concentration increases of Mg^2+^ and Ca^2+^ in generated slurry [45]. Hence, the structural strain of dolomite caused by mechanical grinding is speculated to supply calcium and magnesium in GBM slurry for hydration reaction [26]. Kaolinite and muscovite are aluminosilicate clay minerals [46]. They are phyllosilicates commonly constituted by a layer structure made of sheets of Si-centered tetrahedra alternating with sheets of Al-centered octahedra. As a 1:1 dioctahedral clay, the layers of kaolinite are held together by hydrogen bonds (HBs) between Si-O and Al-OH. As a 2:1 dioctahedral clay, the layers of muscovite are held together by electrostatic forces between the charged layers and interlayer cations such as K^+^, Na^+,^ or Ca^2+^. The destruction of kaolinite crystal structure by mechanical and thermal activation is widely applied to prepare CG-based geopolymer for its moderate content and fragile structure. Therefore, the mechanical grinding converted crystal structures of dolomite, kaolinite, and muscovite into an amorphous phase, which potentially provides free Mg^2+^, Ca^2+^, SiO_2_, and Al_2_O_3_ species in the alkaline slurry.

In Figure 4b, the XRD spectra of CG powder obtained by grinding for 90 min with 1%, 3%, and 5% urea were given. Compared to G0, for G90Uy samples, obvious destruction of dolomite, kaolinite, and muscovite structures also occurred after grinding with urea. However, in contrast with G90, the diffraction intensity of dolomite and kaolinite for G90Uy samples significantly varied with urea dosage. The presence of urea weakened the destroying effect of mechanical grinding on the crystal structure of dolomite. This was consistent with the results of the weaker refining effect on particle size mentioned above. Moreover, for kaolinite structure, the feature peak at 12.4° further decreased with urea addition. Meanwhile, a new peak assigned to the urea/kaolinite intercalation compound (UKC) [47,48,49] appeared at 8.3°. Instead of the original HB interaction between Si-centered tetrahedra and Al-centered octahedra sheets, the urea inserted into the crystal layers with the HB interaction between Si-O of kaolinite and amino group (-NH_2_) of urea, as well as Al-OH of kaolinite and carbonyl group (-CO-) of urea. This intercalation led to the interlayer space increasing from 0.72 nm to 1.08 nm, thus favoring the slip and even deformation of the crystal layer [50].

#### 3.1.3. Chemical Bond

**Figure 5:**
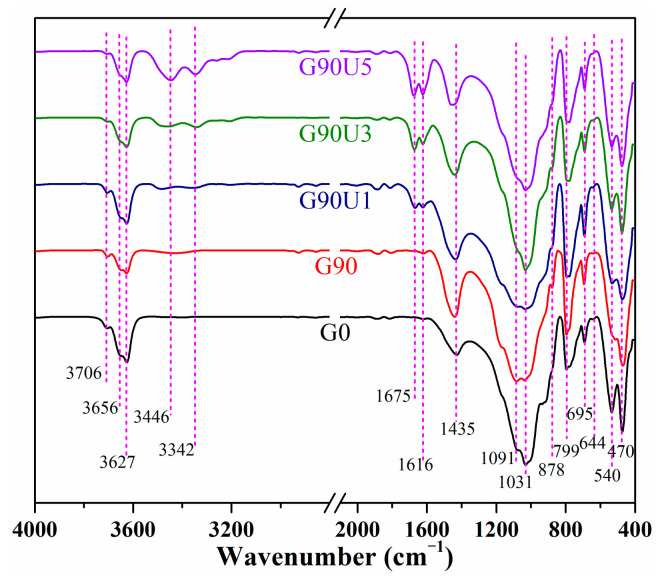
FTIR spectra of G0, G90, G90U1, G90U3, and G90U5.

As depicted in Figure 5, the changes in the chemical bond of CG induced by mechanical grinding for 90 min were analyzed by FTIR spectra. Compared to G0, the spectral changes of G90 and G90Uy were first discussed. The signal at 470 cm^−1^ was assigned to the Si–O–Si symmetric bending vibration. No obvious variation in this signal indicated the considerable retention of stable quartz structure after grinding for 90 min. The signals at 540 cm^−1^ and 430 cm^−1^ were mainly attributed to the bending vibration of Si–O–Al^VI^, while the signal at 878 cm^−1^ was ascribed to a tetrahedral Al^IV^–O structure. The intensity decreases at 540 cm^−1^ and 430 cm^−1^, and the simultaneous increase in 878 cm^−1^ was mainly due to the transformation from Si–O–Al^VI^ to Al^IV^–O caused by mechanical grinding. Moreover, the multiple peaks at 3800~3600 cm^−1^ were assigned to the stretching vibration of inner surface hydroxyl and inner hydroxyl of Al^VI^–O octahedra, while the peaks at 940 and 913 cm^−1^ were ascribed to their bending vibration [25]. Meanwhile, the broadband at 3600~3200 cm^−1^ was related to the HB of water molecules, and the signal at 1616 cm^−1^ was assigned to their bending vibration. The hydroxyl signals weakened, and HB signals enhanced, implying the removal of inner hydroxyl and an increase of absorbed water with grinding. Overall, these variations in chemical bonds suggested the partial deformation of the kaolinite structure, which was also proved by the XRD analysis mentioned above. Additionally, compared with G90, for G90Uy, distinct signals at 3444 and 3344 cm^−1^ related to amino stretching vibration of urea appeared. Another newly generated signal at 1683 cm^−1^ was ascribed to carbonyl that connected with primary amino groups of urea [49].

### 3.2. Transportability of GBM Slurries

For GBM applications, the raw materials are mixed with water and conveniently transported to the filling site as slurries via a pipe equipped with a pump. Therefore, transportability became a major concern in the technique’s design. Herein, the bleeding rate and slump flow were studied to evaluate the transportability of fresh GBM slurry. The bleeding rate (*B*_w_) was determined by the mass ratio of bleeding water to added water in each slurry sample. The *B*_w_ values reflect water retention and precipitation features of solid particles in slurries. Excess bleeding water will increase the unnecessary water consumption and workload of water drainage. Meanwhile, the considerable segregation of solid particles also might result in blockage in pipelines. The practical experience shows that *B*_w_ lower than 5% is an acceptable criterion for the backfilling slurry [28]. Moreover, the slump flow (*D*_f_) was determined by the average diameter of the freely flowing slurry in a slump test conducted with a circular truncated cone (φ36 × φ60 × h60 mm). The *D*_f_ values characterize the capacity of flow and extension for slurries. In general, poor slump flow indicates that the slurry is too viscous. Typically, the GBM slurry demands for excellent fluidity to maintain a steady flow in pipelines and extend swimmingly in the filling space. Hence, referring to the fresh cemented slurry, *D*_f_ larger than 200 mm is regarded as a criterion for grouting slurry with superior flowability.

#### 3.2.1. GBM-A1 Formulation

**Figure 6:**
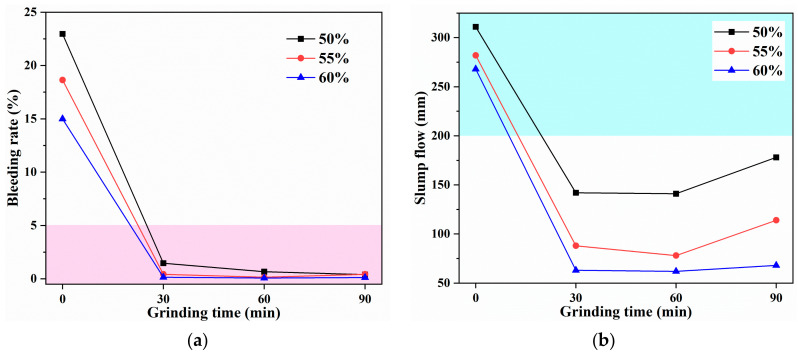
The (**a**) bleeding rate (*B*_w_) and (**b**) slump flow (*D*_f_) of GBM-A1 slurry using CG-A obtained by grinding without additives.

First, the basic GBM formulation using CG-A obtained by grinding without any additives was investigated. CG-A and cement were mixed with water to provide a GBM slurry, which was termed as GBM-A1 series. The CG-A was specifically labeled as Gx, while x represented grinding for x minutes. The mass ratio of CG and cement was fixed at 85:15, and the solid concentration varied from 50% to 55% to 60%. The transportability characteristics, including bleeding rate (*B*_w_) and slump flow (*D*_f_), were displayed in Figure 6. Overall, with the increase of solid concentration, the slurries got lower *B*_w_ and *D*_f_. Therefore, concentration regulation offers a basic approach to regulating the transportability of slurries. For GBM slurry using G0 without grinding treatment, severe water bleeding and solid settling were observed in all cases with a solid concentration of 50~60%. In spite of *D*_f_ values larger than 200 mm, *B*_w_ values of 15~23% proved that raw CG without grinding treatment exhibited a poor capacity for water retention and suspension.

Compared to G0-based slurries, their counterparts using G30, G60, and G90 had much lower *B*_w_ and *D*_f_. The *B*_w_ ranged from 0.12% to 1.5%, while the *D*_f_ ranged from 62 mm to 178 mm. Despite the slight water bleeding, the *D*_f_ values lower than 200 mm showed that the slurries were also far from satisfactory in their smooth transportation. As shown in Figure 6a, the reduction in *B*_w_ was mainly due to the increase in water demand of CG powder with smaller particle size and, thus, larger specific surface area caused by longer grinding duration. The loose and porous CG particles were also reported to absorb more water into pores compared with vitreous spherical fly ash [51]. Meanwhile, as depicted in Figure 6b, the absorption of free water by CG powder inversely hindered the flow and extension of slurries. Notably, compared with slurries employing G30 or G60, the G90-based slurries exhibited slightly larger *D*_f_. It suggested that further refined CG particles with a better capacity of water retention and suspension favored the slurry flow and extension. However, given the energy and time consumption of mechanical grinding, merely prolonging the grinding duration is not an effective and economical strategy to improve slurry transportability in backfilling applications.

#### 3.2.2. GBM-B1 Formulation

**Figure 7:**
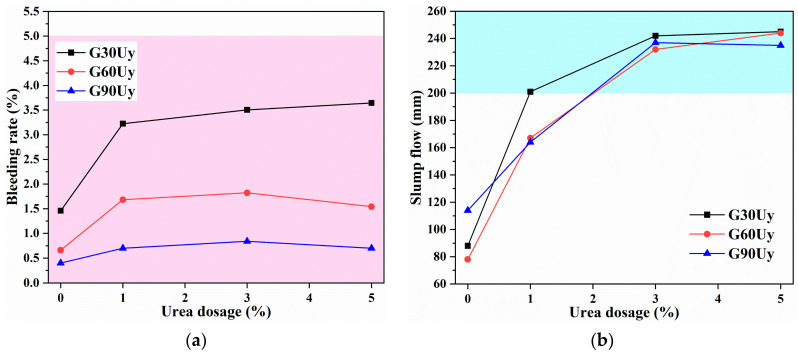
The (**a**) Bleeding rate (*B*_w_) and (**b**) slump flow (*D*_f_) of GBM slurry using CG-B obtained by grinding with urea.

Given the HB mediating effect of urea and its potential to act as a superplasticizer (SP), the GBM formulation utilizing CG-B obtained by grinding with urea was subsequently studied. CG-B and cement were mixed with water to offer a GBM slurry, which was termed as GBM-B1 series. The CG-B was specifically labeled as GxUy, while x and y represented grinding for x minutes with y% urea. As consistent with the GBM-A1 series, the mass ratio of CG and cement was also set as 85:15, while the solid concentration was fixed at 55%. Figure 7a shows the bleeding rate (*B*_w_) evolution of slurries based on GxUy with urea dosage varying from 0 to 5%. The addition of urea obviously increased the *B*_w_, whereas overall *B*_w_ values still remained lower than 5%. A comparison with GxU1, GxU3, and GxU5 exhibited no significant variation in *B*_w_ with an increment of urea. It suggested that a urea dosage of 1% is sufficient to release the freest water encapsulated in hydrated cement particles mainly by HB interaction between urea and cement particles. Moreover, as listed in Table 2, the presence of urea resulted in a reduction in the surface tension of the aqueous phase. The surface tension declined from 73 to 39 mN/m as urea dosage varied from 0 to 3%. This change improved the surface wettability of CG particles [52], decreased the water absorption on their surface, and thus, further released free water in slurry.

Meanwhile, Figure 7b displays slump flow (*D*_f_) variation of slurries based on GxUy with urea dosage changing from 0 to 5%. Corresponding to *B*_w_ trends, the *D*_f_ increased as well with urea addition. It indicated that the release of free water due to the HB mediating effect by urea also benefited the slurry flow and extension. G30Uy, G60Uy, and G90Uy with urea dosages of 3~5% got maximal *D*_f_ values of about 232~245 mm. In comparison with G60U1 and G90U1, G30U1, with a larger particle size analyzed in Figure 3b, also contributed an acceptable *D*_f_ of 201 mm. Therefore, G30U1, GxU3, and GxU5 (x = 30, 60, 90) offered stable and smooth slurries satisfying transportability criteria concerning both *B*_w_ and *D*_f_.

#### 3.2.3. GBM-A2 and GBM-B2 Formulations

**Figure 8:**
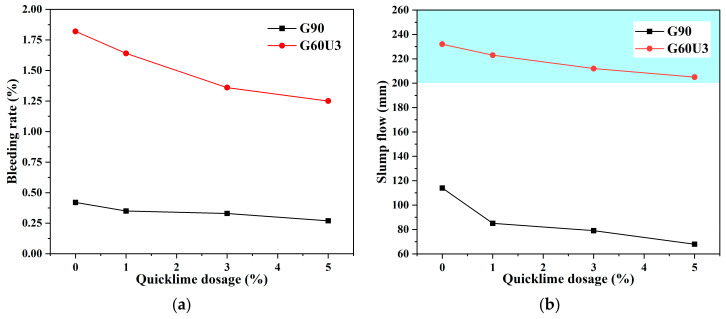
The (**a**) Bleeding rate (*B*_w_) and (**b**) slump flow (*D*_f_) of GBM slurry using G90 and G60U3 with the addition of quicklime.

The mechanically activated CG was reported to participate in a pozzolanic reaction by incorporating calcium hydroxide (CH). Hence, for CG-based GBMs in this section, the quicklime (CaO) was further employed as an additive to supply CH via a simple hydration reaction. For example, G90 and G60U3 were selected to explore the effect of quicklime on the transportability of GBM slurries. G90, cement, and quicklime were mixed with water to provide a GBM slurry, which was termed as GBM-A2 series. Meanwhile, G60U3, cement, and quicklime were mixed with water to offer a GBM slurry, which was termed as GBM-B2 series. As consistent with the GBM-A1 and GBM-B1 series, the mass ratio of CG and cement was still fixed at 85:15, while the solid concentration was also set at 55%. The quicklime dosage was calculated by the mass ratio of quicklime to CG and varied in a range from 1% to 5%. The transportability results, including bleeding rate (*B*_w_) and slump flow (*D*_f_), are shown in Figure 8.

In Figure 8a, compared to G90-based GBM-A1 and G60U3-based GBM-B1 slurries, the *B*_w_ of GBM-A2 and GBM-B2 counterparts decreased with quicklime addition. This variation was mainly due to the water consumption of quicklime by a hydration reaction. As quicklime dosage increased from 1% to 5%, the *B*_w_ of G90-based GBM-A2 decreased from 0.35% to 0.27%, while the *B*_w_ of G60U3-based GBM-B2 reduced from 1.64% to 1.25%. For inherent water absorption of CG powder and additional water consumption of quicklime, the *B*_w_ values in all cases herein were lower than 5%. However, in Figure 8b, the water reduction caused by quicklime addition inversely decreased the *D*_f_. For G90-based GBM-A2, with quicklime dosage increasing from 1% to 5%, the *D*_f_ declined from 85 mm to 68 mm. Moreover, for G60U3-based GBM-B2, the *D*_f_ decreased from 223 mm to 205 mm with a quicklime dosage of 1~5%. Despite additional water consumption of quicklime, G60U3-based GBM-B2 slurries with *D*_f_ larger than 200 mm were still attained. This also proved that the HB mediating effect of urea favored the slurry flow and extension in the presence of quicklime. Therefore, G60U3-based GBM-B2 formulation with a quicklime dosage of 1~5% provided stable and smooth slurries meeting transportability criteria considering both *B*_w_ and *D*_f_.

### 3.3. UCS of Hardened GBM Specimens

**Figure 9:**
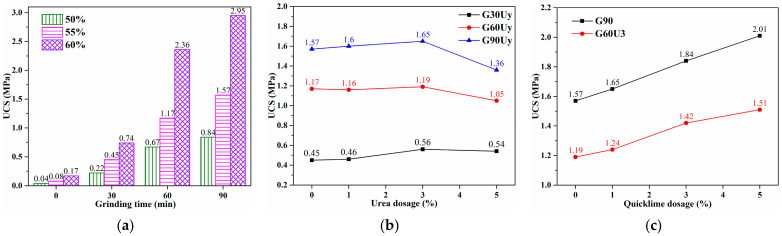
The UCS of Gx-based GBM-A1 (**a**), GxUy-based GBM-B1 (**b**), G90-based GBM-A2, and G60U3-based GBM-B2 (**c**) specimens after curing for 7 days.

The GBM slurry finally formed a hardened body via bleeding water and consolidation to provide structural support in the filling site. The uniaxial compressive strength (UCS) was typically employed to evaluate the mechanical performance of the hardened body. For grouting materials [9,53], seven-day UCS is an essential parameter to evaluate their strength performance. Herein, the UCS of hardened GBM specimens after curing for seven days was studied. The UCS evolutions with grinding time of CG powder, urea, and quicklime dosage were depicted in Figure 9.

#### 3.3.1. GBM-A1 Formulation

Figure 9a shows the UCS results of basic GBM-A1 formulation using CG-A (specifically labeled as G30, G60, and G90) obtained by grinding without any additives. In accordance with transportability studies, the mass ratio of CG and cement was fixed at 85:15, and the solid concentration varied from 50% to 55% to 60%. After curing for seven days, G0-based GBM specimens got relatively low UCS of 0.04~0.17 MPa. The relatively low compressive strength can be explained from two aspects. On the one hand, compared with coarse aggregate with a diameter greater than 5 mm, the fine grit of raw CG powder supplies a weaker constructive support. On the other hand, compared with supplementary cementitious materials (SCMs), such as fly ash and calcined CG, the inert property of raw CG powder contributes little to the cementitious matrix. Therefore, besides the severe water bleeding of slurries discussed in transportability studies [Figure 6a], the UCS of hardened bodies based on G0 without mechanical grinding was far from the strength requirements in most backfilling applications.

With grinding time increasing from 30 to 90 min, GBM specimens using G30, G60, and G90 got much higher UCS of 0.22~0.74 MPa, 0.67~2.36 MPa, and 0.84~2.95 MPa, respectively. It indicated that mechanical grinding potentially enhanced the pozzolanic activity of CG powder. The pozzolanic reaction between the active Si/Al phase in CG powder and CH from cement hydration generated hydrated calcium silicate (C–S–H) gel, which further strengthened the hardened bodies [28]. Moreover, with the increase of solid concentration, the slurries universally got higher UCS. This mainly benefited from the denser stacking structure and faster hydration reactions caused by the higher solid concentration. Therefore, concentration regulation also offers an efficient strategy for regulating the mechanical performance of hardened GBMs.

Furthermore, two-way ANOVA was performed using Microsoft Office Excel (alpha = 0.05), and the results were listed in Table 3. Statistically significant differences (*p* < 0.05) were detected both for the solid concentration of GBM slurry (*p* = 0.043) and for the grinding time of CG powder (*p* = 0.016). This also proves that increasing solid concentration and prolonging grinding duration offer effective strategies to improve the compressive strength of GBMs.

#### 3.3.2. GBM-B1 Formulation

Figure 9b displays the UCS evolution of GBM-B1 formulation utilizing CG-B (specifically labeled as G30Uy, G60Uy, and G90Uy) obtained by grinding with urea. As consistent with transportability studies, the mass ratio of CG and cement was also set as 85:15, while the solid concentration was fixed at 55%. With urea dosage of 0~3%, slight variations appeared in UCS of G30Uy, G60Uy, and G90Uy-based GBM specimens within 1.57~1.65 MPa, 1.16~1.19 MPa, and 0.45~0.56 MPa, respectively. This implied that the urea addition had a negligible effect on strength development, although intercalation of urea into limited content of kaolinite occurred during CG grinding, as discussed in Figure 4b. Furthermore, with a higher urea dosage of 5%, obvious decreases in UCS appeared for G60Uy and G90Uy GBMs. This suggested that further higher urea dosage inversely hindered strength development, which might be caused by both weakened refining effect on CG particles and delaying effect on the cement hydration [34].

#### 3.3.3. GBM-A2 and GBM-B2 Formulations

Figure 9c shows the UCS variation of G90-based GBM-A2 and G60U3-based GBM-B2 formulations with quicklime dosage. As consistent with transportability studies, the mass ratio of CG and cement was still fixed at 85:15, while the solid concentration was also set at 55%. For the G90-based GBM series, the UCS increased from 1.57 MPa without quicklime addition to 1.65~2.01 MPa with a quicklime dosage of 1~5%. However, the poor flowability of G90-based GBM-A2 slurries [Figure 8b], mainly caused by the inherent water absorption of G90 powder and additional water consumption of quicklime, made it adverse to their backfilling applications. Meanwhile, for the G60U3-based series, the UCS increased from 1.19 MPa without quicklime addition to 1.24~1.51 MPa with a quicklime dosage of 1~5%. The improvement in UCS could be explained by an accelerated pozzolanic reaction between CG powder and additional CH supplied by the quicklime hydration [26]. In other words, the quicklime addition helps to compensate for the UCS loss of GxUy-based GBMs in comparison with Gx-based counterparts. Moreover, despite additional water consumption of quicklime, the G60U3-based GBM-B2 slurries still maintained excellent transportability, verified in Figure 8. Therefore, the incorporation of urea and quicklime as an optimizing strategy has been demonstrated to offer CG-based GBMs with both transportability and strength acceptable to grouting backfilling applications.

## 4. Comprehensive Evaluation of CG-Based GBMs

### 4.1. Activation Efficiency of Ball Grinding

Ball grinding for 30~90 min effectively decreased CG particle size, with Da declining from 89.6 μm to 5.8~8.1 μm, and even induced obvious crystal deformation of dolomite and kaolinite. For CG-based GBMs, a statistically significant difference (Table 3, *p* < 0.05) was detected for the grinding time of CG powder. It also proves that mechanical grinding offers an effective strategy to improve the reactivity of CG powder and, thus, the compressive strength of GBMs. However, urea addition during grinding weakened the refining effect on particle size and had a destructive effect on minerals.

### 4.2. Workability and Strength of GBM

(1) GBM using raw CG power (G0) with a solid content of 85% at 50~60 wt% exhibited excess bleeding water of 15~23% and inferior seven-day UCS of 0.04~0.17 MPa. It shows that raw CG without grinding treatment has a poor capacity for water retention and suspension. Moreover, fine grit, as well as the inert property of raw CG powder, are beneficial for neither constructive support nor cementitious matrix.
(2) GBM-A1 using Gx obtained by grinding had a reduced slump flow of 62~178 mm and improved seven-day UCS of 0.22~2.95 MPa. Meanwhile, G90-based GBM-A2 with quicklime addition appeared to further decline slump flow and improve UCS. The decline in slump flow suggests that increased water absorption by refined grit and additional water consumption by quicklime hydration hinder slurry flow and extension. The strength increment could be interpreted by the enhanced reactivity of ground CG powder to offer cementitious products via a pozzolanic reaction with CH supplied by cement and quicklime hydration.
(3) GBM-B1 using GxUy prepared by grinding with urea of 3~5% exhibited excellent transportability with bleeding water below 5% and maximal slump flow of 232~245 mm. It is possibly due to decreased water absorption on the CG surface and the release of water encapsulated in hydrated cement particles. The urea/kaolinite intercalation compound (UKC) formed via HB interaction during grinding, leading to an increase in interlayer space, favoring the slip of the crystal layer and, thus, the rheological property of GBM slurry. However, urea appeared to have a negligible or even adverse effect on strength development. This might be caused by both the weakened refining effect on CG particles and the delaying effect on cement hydration.
(4) G60U3-based GBM-B2 with 5% quicklime provided a stable and smooth slurry with a bleeding rate of 1.25%, a slump flow of 205 mm, and a hardened body with a seven-day UCS of 1.51 MPa. The quicklime addition helps to compensate for the UCS loss of GxUy-based GBMs in comparison with Gx-based counterparts.

In this work, the GBMs were designed for backfilling in the mining-induced overburden bed separation and mined-out area with caving rocks to control strata movement caused by underground mining [5]. In engineering applications, under the pressure of overlying strata, a compacted body with broken rocks is finally formed via bleeding water and consolidation of grouting slurry to offer support in the filling site. The final support effects are synthetically influenced by various factors, such as filling ratio, crack development, and so forth. Currently, there are no strict requirements for the strength of hardened specimens formed without pressure. Referring to a similar study [9], after a 28-day standard curing period, the compressive strength should be more than 1 MPa. A grouting material with cement, fly ash, and slag powder of 20:30:50 has a three-day strength of 5 MPa [27]. A bentonite-based grouting material with 40% cement has a seven-day strength of 0.4~2 MPa [9]. Therefore, the seven-day UCS of 1.51 MPa for G60U3-based GBM-B2 with 5% quicklime is acceptable for backfilling. The CG-based GBMs are expected to supply an alternative to those relying heavily on fine aggregates such as FA, clay, or cement.

### 4.3. Economic Cost

The G60U3-based GBM-B2 with 5% quicklime was taken as an example to analyze the economic cost of grinding and incorporation of additives. The electric cost of grinding and the unit cost of materials are listed in Table 4. As calculated in Equation (1), the cost roughly consists of CG grinding, cement, urea, and quicklime:

0.85 × 168 + 0.15 × 450 + 0.85 × 3% × 2100 + 0.85 × 5% × 320
= 142.8 + 67.5 + 53.55 + 13.6 = 277.45 (RMB/t)(1)

The economic cost of targeted GBM is 277.45 RMB/t. This is relatively lower than pure cement GBM, with a unit cost of 450 RMB/t. Notably, the electric cost of grinding accounts for 51% of the total cost. Meanwhile, the cost of additives, including urea and quicklime, occupies 25%, which spends roughly the same share as cement, at 24%. Therefore, in order to save the filling cost of CG-based GBMs, more efficient methods for mechanical activation of CG, such as wet-grinding with fewer additives, call for further studies.

## 5. Conclusions

Cemented GBM formulations using ground CG powder as a dominant component were studied in this work. The incorporation of urea and quicklime as additives to optimize slurry transportability and compressive strength was demonstrated. The conclusions can be drawn as follows:

(1) Mechanical grinding under ambient conditions supplies an effective pathway to activate CG for its disposal and functional applications. It was found that ball grinding for 30~90 min effectively decreased CG particle size and even induced obvious crystal deformation of dolomite and kaolinite.
(2) The incorporation of urea and quicklime provides a promising strategy to offer CG-based GBMs with both transportability and strength acceptable to grouting backfilling applications in mining-induced overburden bed separation or mined-out areas with caving rocks. G60U3-based GBM-B2 with 5% quicklime provided a stable and smooth slurry with a bleeding rate of 1.25%, a slump flow of 205 mm, and a hardened body with a seven-day UCS of 1.51 MPa.

GBM formulations with a principal content of mechanically ground CG are expected to supply an alternative to those relying heavily on fine aggregates, such as FA, clay, or cement. In this work, we paid more attention to slurry fluidity to demonstrate that urea is an alternative superplasticizer but didn’t significantly increase strength or lower economic cost. Therefore, optimization of GBM strength and cost needs further investigations concerning more parameters with lower requirements on slurry fluidity.

## Figures and Tables

**Table 1:** Mix ratio of raw materials to prepare GBM specimens.

GBM Series	CG Powder ^1^	Solid Concentration (wt%) ^2^	Urea Dosage (%) ^3^	Quicklime Dosage (%) ^4^
Control group	G0	50, 55, 60	0	0
GBM-A1	G30	50, 55, 60	0	0
	G60	50, 55, 60	0	0
	G90	50, 55, 60	0	0
GBM-A2	G90	55	0	1, 3, 5
GBM-B1	G30Uy	55	1, 3, 5	0
	G60Uy	55	1, 3, 5	0
	G90Uy	55	1, 3, 5	0
GBM-B2	G60U3	55	3	1, 3, 5

^1^ The mass ratio of CG powder and cement was fixed as 85:15. ^2^ The solid concentration was determined by mass ratio of [CG + cement]/[CG + cement + water]. ^3^ The urea dosage was determined by mass ratio of urea to CG. ^4^ The quicklime dosage was determined by mass ratio of quicklime to CG.

**Table 2:** The surface tension of deionized water and urea solution.

Urea Dosage ^1^	0	0.5%	1.0%	3.0%
Surface tension (10^−3^ N/m)	73	60	55	39

^1^ The urea dosage of the aqueous solution was determined by the mass ratio of [urea]/[water].

**Table 3:** Two-way ANOVA results for solid concentration of GBM slurry and grinding time of CG powder.

Source of Difference	SS	df	MS	F	*p*-Value	F Crit
Solid concentration	2.562917	2	1.281458	5.548973	0.043214	5.143253
Grinding time	5.581633	3	1.860544	8.056533	0.015868	4.757063
Error	1.385617	6	0.230936			
Total	9.530167	11				

**Table 4:** Economic cost involved for G60U3-based GBM-B2 with 5% quicklime.

Expenditure Item	Unit Cost (RMB/t)	Individual Cost (RMB for 1 t GBM)	Individual Cost Proportion
Grinding for 60 min	168 [22]	142.8	51%
Cement	450	67.5	24%
Urea	2100	53.55	19%
Quicklime	320	13.6	6%

## Data Availability

Data is contained in the article.

## Abbreviations

CG	coal gangue
Da	average diameter
DN	diameter when cumulative distribution reaching N%
FTIR	Fourier transform infrared spectroscopy
GBM	grouting backfill material
HB	hydrogen bonding
SP	superplasticizer
UCS	uniaxial compressive strength
XRD	x-ray diffraction
Gx	CG powder obtained by grinding for x min without admixtures
GxUy	CG powder obtained by grinding for x min with y% urea
CH	calcium hydroxide [Ca(OH)_2_]
